# Description of *Prionchulus jonkershoekensis* n. sp. (Nematoda: Mononchida), a new predatory species from South Africa

**DOI:** 10.21307/jofnem-2021-039

**Published:** 2021-03-26

**Authors:** Candice Jansen van Rensburg, Hendrika Fourie, Samad Ashrafi, Milad Rashidifard

**Affiliations:** Department of Zoology and Entomology, University of the Free State, P.O. Box 339, Bloemfontein, 9300, South Africa; Unit for Environmental Sciences and Management, Integrated Pest Management, North-West University, Private Bag x6001, Potchefstroom, 2520, South Africa; Julius Kühn-Institut (JKI), Federal Research Centre for Cultivated Plants, Institute for Epidemiology and Pathogen Diagnostics, Messeweg 11-12, 38104 Braunschweig, Germany

**Keywords:** Mononchida, Morphology, Morphometrics, Ribosomal DNA, Taxonomy

## Abstract

*Prionchulus jonkershoekensis* n. sp. is described from South Africa and illustrated using morphological, morphometric, and molecular techniques. This species is characterized by its body length (1.78–2.14 mm); the size of buccal cavity (38–44 × 24–31 µm), lower dorsal tooth position in relation to buccal cavity base, the position of amphidial aperture just above dorsal tooth apex, *pars proximalis vaginae* with almost straight walls, and tail 144–158 µm long with sickle shaped posterior third part. Phylogenetic analyses based on 18 S rDNA and 28 S rDNA of *P. jonkershoekensis* n. sp. revealed close relationships of the new species with *Prionchulus punctatus* and *Prionchulus muscorum*. This is also an additional geographical record for the genus from South Africa.

Species belonging to the genus *Prionchulus* Cobb, 1916 are predatory nematodes and have been described from terricolous and limnic habitats (Andrássy, 2006). Members of the genus are easily recognizable; however, species within the genus share high morphological similarities (Vu et al., 2018) making identification problematic. The genus has been revised several times by many taxonomists (Mulvey, 1967; Andrássy, 1985, 1993), with Winiszewska and Susulovsky (2003, 2004) also redescribing a number of species from type material. Species lists of the *Prionchulus* have been revised and updated by different scientists in the last decade (Andrássy, 2006; Ahmad and Jairajpuri, 2010; Jana et al., 2010). Members of the family Mononchidae, to which the genus *Prionchulus* belongs, have been poorly studied in South Africa. Only one species, *Prionchulus muscorum* (Dujardin, 1845) Cobb, 1916 has previously been reported from the Kowynspas (Coetzee, 1968) in the Gauteng Province, South Africa.

The genus has a cosmopolitan distribution, with Europe being particularly rich in species (Andrássy, 2006). To date, 30 species have been described from this genus worldwide (Kim et al., 2018). Most of the species in the genus have been collected from moss, leaf litter, and forest soil (Susulovsky and Winiszewska, 2002; Winiszewska and Susulovsky, 2003; Orselli and Vinciguerra, 2007) associated with riverbanks and mountainous regions (Susulovsky and Winiszweska, 2006; Farahmand et al., 2009).These predatory nematodes play an important role in bioregulation of litter and soil communities (Dipchikova et al., 2019). 

During a survey of the Jonkershoek Mountains in Stellenbosch, South Africa, samples were collected from seven localities to study nematodes associated with leaf litter. Among the various nematodes extracted, one population of *Prionchulus* was collected. A thorough investigation revealed that this was an undescribed species, and is described herein as *Prionchulus jonkershoekensis* n. sp. This species was characterized using both morphological and molecular techniques, its phylogenetic relationships are also discussed based on 18 S and 28 S rDNA genes.

## Materials and methods

### Nematode extraction and processing

*Prionchulus jonkershoekensis* n. sp. specimens were extracted from leaf-litter samples collected from one locality in the Jonkershoek Mountains by using the Baermann tray method (Hooper and Evans, 1993). Nematodes were heat-killed and fixed using the glycerol-ethanol method of Seinhorst (1959) as modified by De Grisse (1969), and mounted on Cobb slides in anhydrous glycerin for identification purposes. Specimens for molecular analysis were stored in DESS solution (dimethyl sulphoxide, disodium EDTA, and saturated NaCl).

Measurements and drawings of mounted *Prionchulus* specimens were made with the aid of a Nikon 80i microscope equipped with a drawing tube. Micrographs were taken with an automatic camera system mounted (Axiocam ICc 5) on Zeiss Axiophot microscope. Using the drawings made with a drawing tube as a template, digital drawings were constructed in CorelDRAW X5.

### Scanning electron microscopy

Mounted specimens were removed carefully from slides, hydrated in distilled water, dehydrated in a graded ethanol series, critical point dried, and coated with gold (Green, 1967), and observed under a JEOL JSM-7800F Field Emission Scanning Electron Microscope at 5 kV.

### Molecular characterization

Genomic DNA was extracted from a single female specimen using the modified Chelex method as described by Rashidifard et al. (2019). The DNA was amplified using the following reagents: 12.5 µl master mix (Promega), 4 µl of specimen’s DNA, 1 µl of forward and reverse primers (10 µM), and 6.5 µl of ddH2O in a Vacutec thermocycler machine (www.vacutec.co.za). Two nuclear loci were amplified and sequenced: partial 18 S rDNA (SSU) using primers SSU F04 (5’–GCTTGTCTCAAAGATTAAGCC–3’), and SSU R26 (CATTCTTGGCAAATGCTTTCG) (Blaxter et al., 1998); partial 28 S rDNA (LSU) using D2A (5’–ACAAGTACCGTGAGGGAAAGTTG–3’), and D3B (5’–TCGGAAGGAACCAGCTACTA–3’) (Subbotin et al., 2006). The PCR program used for amplification was as follows: DNA initial denaturation at 94°C (3 min), 35 cycles of denaturation at 94°C (45 s), annealing at 54˚C (SSU) and 56°C (LSU) (45 s), extension at 72°C (45 s), and a final extension at 72°C (6 min). PCR products were sequenced in both forward and reverse directions by Inqaba biotec™ (South Africa; www.inqaba-southafrica.co.za).

### Phylogenetic analyses

The newly generated sequences were aligned using MUSCLE alignment tool (Edgar, 2004) in Geneious Prime 2020.2.3 (www.geneious.com) and consensus sequences were extracted and used for further analyses. The newly obtained consensus sequences of 18 S and 28 S rDNA genes were compared to those available for other species in Genbank using BLAST search. Two data sets were prepared for 18 S and 28 S genes using available mononchid sequences in Genbank; newly obtained sequences were also included. Both 18 S and 28 S rDNA datasets were aligned using MUSCLE (Edgar, 2004) in Geneious Prime 2020.2.3 (www.geneious.com). The General Time Reversible model with a Gamma distribution (GTR + G) was selected as the most appropriate nucleotide substitution model for both data sets according to jModelTest 2.1.10 (Darriba et al., 2012).

Bayesian inference (BI) analyses were conducted using MrBayes v3.1.2 (Ronquist and Huelsenbeck, 2003) implemented in Geneious Prime 2020.2.3. Two Markov chain Monte Carlo (MCMC) were run from a random starting tree for 2 million generations and trees were sampled every 100 generations. Burn-in sampled trees (25%) were discarded. The remaining trees were used to calculate the 50% majority rule consensus tree using the MCMC algorithm to estimate the Bayesian posterior probabilities (BPP) (Larget and Simon, 1999).

## Results

### Systematics

**Figure 1:**
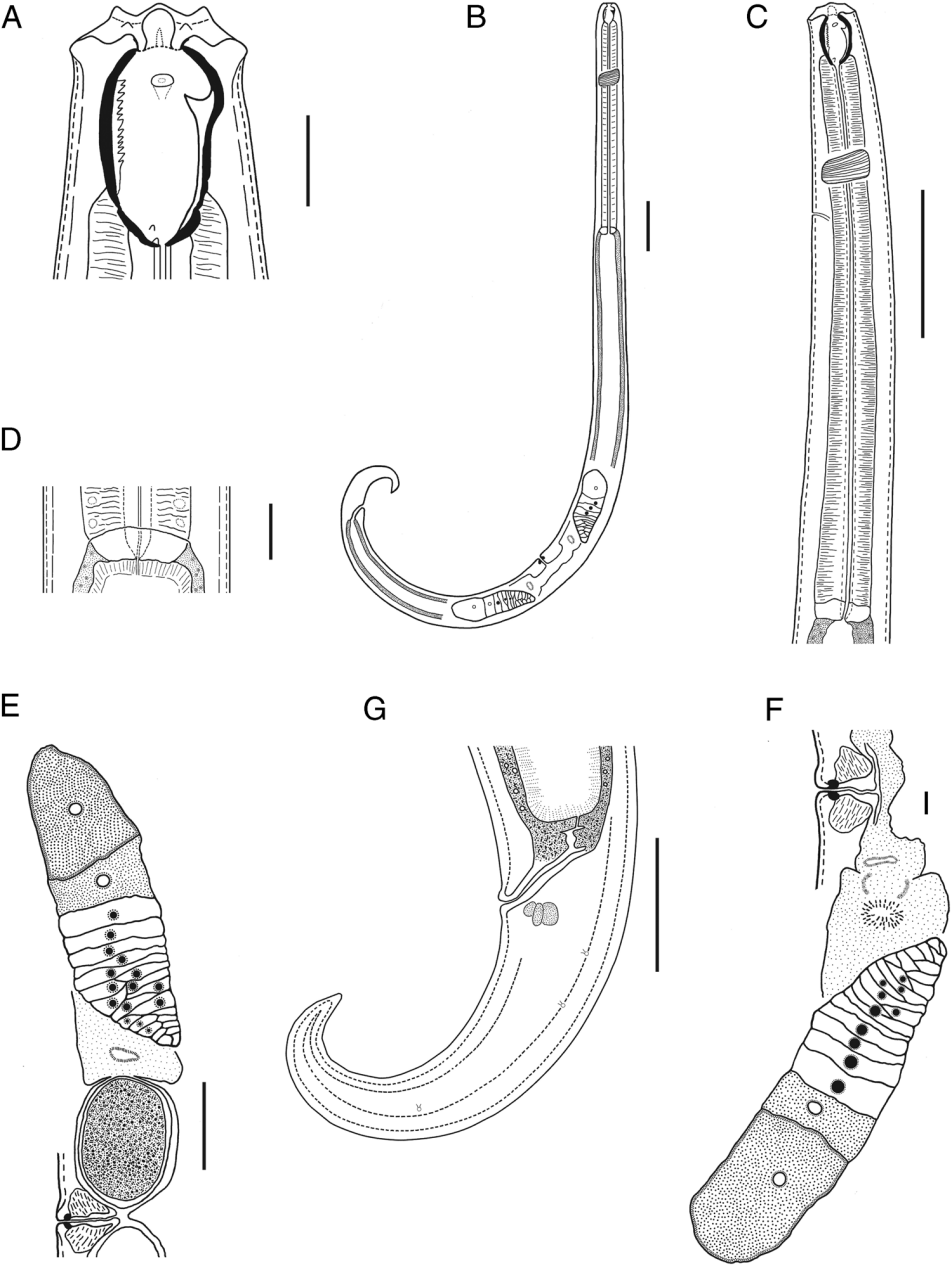
Line drawings of *Prionchulus jonkershoekensis* n. sp. female. (A) Head region in lateral view; (B) Entire body; (C) Neck region; (D) Pharyngo-intestinal junction (cardia); (E) Anterior genital branch with egg; (F) Posterior genital branch; (G) Rectal region and tail. (Scale bars: A, D = 20 µm; B, C = 100 µm; E, G = 50 µm; F = 10 µm).

**Figure 2:**
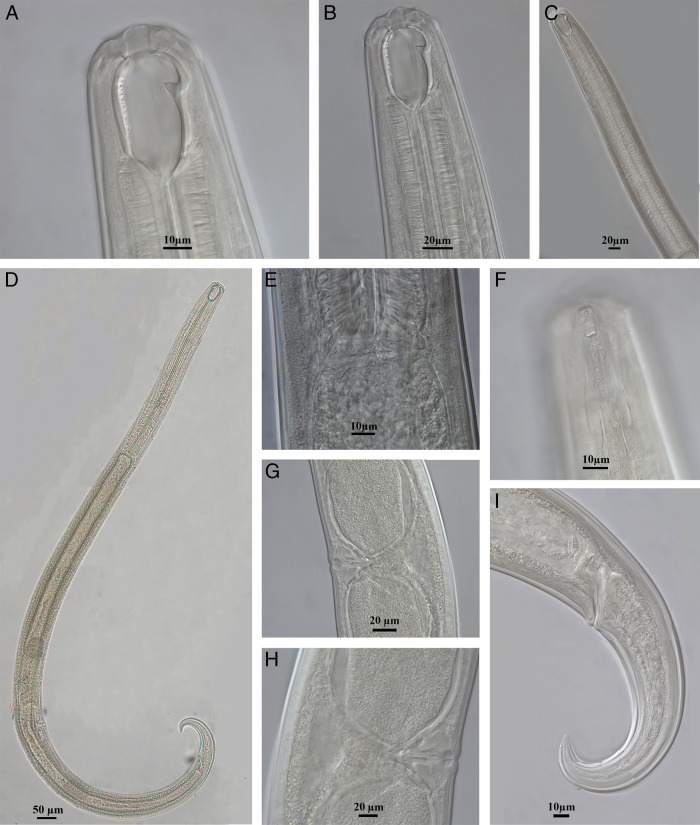
Light micrographs of *Prionchulus jonkershoekensis* n. sp. female. (A-B) Anterior region in lateral median view; (C) Neck region; (D) Entire body; (E) Pharyno-intestinal junction; (F) Laterial lip region showing amphidial fovea and cuticle striations; (G-H) Vagina; (I) Caudal region.

**Figure 3:**
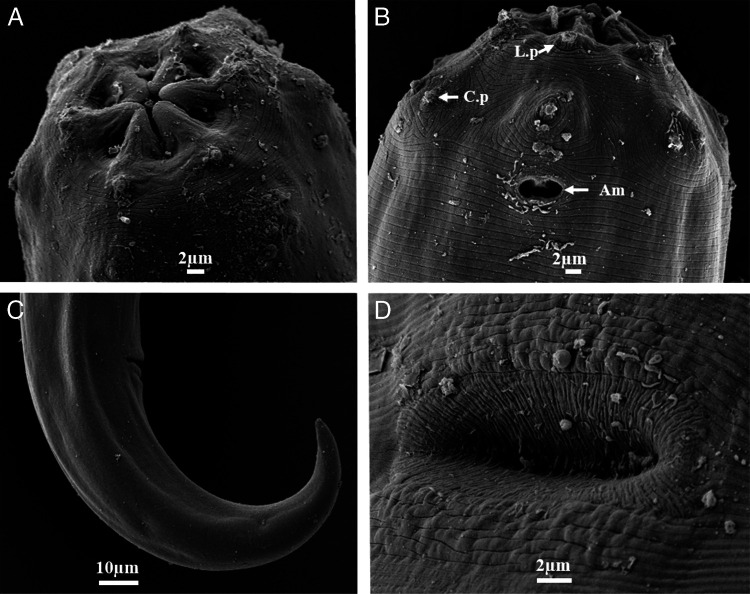
Scanning electron micrographs of *Prionchulus jonkershoekensis* n. sp. female. (A) Lip region (Frontal view); (B) Lateral view showing labial and cephalic papillae and amphidial fovea; (C) Tail; (D) Vulva (ventral view). (L.p- Labial papillae, C.p- Cephalic papillae, Am- Amphidial fovea).

*Prionchulus jonkershoekensis* n. sp. (Figs. 1–3; Table 1).

### Description

Female: Slender to moderately slender (a = 23–30) nematodes, 1.78–2.14 mm long. Body cylindrical, tapering toward tail terminus. Habitus ventrally curved at posterior end upon fixation, J-shaped. Cuticle moderately thick, with two layers, outer layer thinner than inner layer, with fine transverse striations. Cuticle appears more thickened at vulva and caudal region (2–4 µm). Lateral chord 27–38% of midbody diameter. Body pores obscure. Lip region rounded, offset by weak depression but of similar width with adjacent body, about 3–4 times as wide as high. Lips amalgamated, rounded to angular. Labial papillae slightly more conical and protruding than cephalic papillae.

Amphidial fovea cup-shaped, opening (oval to ellipsoid) at level of anterior denticle above dorsal tooth apex, 3–6 µm in diameter and occupying 1/5–1/6 of lip region width. Stoma consists of a vestibulum and a buccal cavity 1.4–1.7 times as long as wide, with 2–4 µm thick walls and tapering at its base, funnel shaped. Dorsal tooth of medium size situated in lower anterior half of buccal cavity, its apex sharply pointed, located 30–34 µm or 75–81% of the buccal cavity length from its base, its upper edge slightly curved, directed forward. Subventral denticles present, 11–14 in number, relatively large, orientated anteriorly, separated, arranged irregularly in some specimens. Two small foramina observed. Pharynx muscular, cylindrical, about 8–10 times as long as wide, surrounding basal part of stoma. Pharyngeal gland nuclei and outlets indistinct. Nerve ring located at 29–33% of the neck length. Secretory-excretory pore visible, situated 30–38% of the neck length. Cardia oval to rounded, smooth, 0.3–0.5 times as long as wide, 0.2 times as long as body diameter at neck base, non-tuberculate. Intestine uniformly granulated; inner surface lined by short rod-like structures (bacillary layer).

Genital system didelphic-amphidelphic. Genital branches almost symmetrical; anterior branch 300–390 µm and posterior branch 252–362 µm long. Ovaries reflexed, well developed, similar; anterior ovary 124–218 µm and posterior ovary 124–186 µm long; not reaching oviduct-uterus junction. Oviduct with well-marked *pars dilatata,* 40–62 µm in width. Uterus and oviduct separated by a moderate sphincter constriction. Uterus thick-walled, bipartite, 0.5–1.0 times as long as body diameter. Sperm absent. Vagina extending over 33–47% of body diameter. *Pars distalis vaginae* short 4–6 µm long with rounded walls. *Pars refringens vaginae* with well-developed teardrop-shaped sclerotized pieces, with combined width of 10–14 µm, extent along the lumen of 6–8 µm*. Pars proximalis vaginae* 14–22 µm long, longer than wide, with almost straight contours proximally but curved outwards distally, surrounded by circular muscles. Vulva slit transverse, with lips slightly protruding. Eggs ovoid, smooth egg shell. Rectum 36–48 µm long, mostly shorter than anal body diameter. Three unicellular rectum glands present.

Tail conical, 144–158 µm long, bent ventrally, distal 1/3 more curved ventrad, sickle shaped, tapering to finely pointed terminus. Hyaline portion 7 µm long, 5% of tail length. Caudal glands absent. Three pairs of caudal pores present.

Males: Not found.

### Type locality and habitat

The type material was collected from leaf litter and moss near the First Waterfall hiking trail in the Jonkershoek mountains, Stellenbosch, South Africa (coordinates 33°59ʹ54.9ʺ S, 18˚58ʹ59.7ʺ E).

### Type material

Holotype female (slide 51180), and one female and three juvenile paratypes (slide 51181) deposited in the National Collection of Nematodes (NCN), Agricultural Research Council-Plant Health and Protection (ARC-PHP), Pretoria, South Africa; 23 female paratypes deposited in the nematode collection of the University of the Free State (UFS), Bloemfontein, South Africa.

### Etymology

The specific epithet refers to the geographical origin of the new species in the Jonkershoek Mountains in South Africa.

### Diagnosis and relationships

*Prionchulus jonkershoekensis* n. sp. is characterized by females having medium body length (1.78–2.14 mm); labial papillae conical, slightly larger than cephalic papillae; buccal cavity length 38.0–44.0 µm, buccal cavity less than 1.7 times as wide as high; *pars proximalis vaginae* with almost straight walls; *pars refringens vaginae* with teardrop-shaped sclerotized pieces with smooth surface; tail 144–158 µm long, with terminal part ventrally curved, sickle shaped. Moreover, the novelty of the species was confirmed based on sequence of both 18 S and 28 S rDNA genes that revealed enough differences to the other closely related species (*P. muscorum* and *Prionchulus punctatus* (Cobb, 1917) Andrássy, 1958): The new species is molecularly diagnosed from previously described *Prionchulus* species based on 18 S rDNA and 28 S rDNA loci. It differs by 6–16 bp differences from *P. muscorum* in the 18 S rDNA (accession numbers AJ966500 and AY284745, respectively) and by 16–18 bp differences from *P. punctatus* in the 18 S rDNA gene (accession numbers AY284746 and AY284747, respectively). The new species also differs from *P. punctatus* by 60 bp of the 28 S rDNA gene (accession number MG994945). Due to lack of 28 S rDNA sequences of *P. muscorum* in public sequence databases, this species was not included in the phylogenetic analysis of that gene.

The combination of the above-mentioned characters differentiates *P. jonkershoekensis* n. sp. from other species of the genus. *Prionchulus jonkershoekensis* n. sp. resembles *P. muscorum*, *P. hygrophilus* Susulovsky and Winiszewska, 2002, and *P. punctatus* in size, lip region rounded and offset by slight depression, position of the vulva (V = 59–67%), and an *a*-value (*a* = 23–29). However, it differs from *P. punctatus* in the number of subventral denticles (11–14 vs. 16), *b*-value (*b* = 3.0–3.5 vs. 3.6–3.9), neck length (580–610 vs. 360–475 µm), teardrop shaped *pars refringens vaginae* vs. triangular, tail sickle shaped strongly curved ventrad with finely pointed terminus vs. plump tail with broadly rounded tip, and egg surface smooth vs. echinulate; from *P. hygrophilus* it differs in buccal cavity length (38–44 vs. 48–49 µm), buccal cavity length to width ratio 1.4–1.7 vs. 1.8–2.0, vulval lips protruding vs. not protruding, c-value (c = 12–14 vs. 10–12), c’-value (c’ = 2.9–3.5 vs. 4.2–5.0), and shorter tail (144–158 vs. 173–207 µm); from *P. muscorum* it differs by smaller body size [1.78–2.14 vs. 2.50–3.50 mm (Cobb, 1917) vs. 2.29–3.09 mm (Winiszewska and Susulovsky, 2003) vs. 1.45 –3.00 mm (Ahmad and Jairajpuri, 2010)], labial papillae slightly larger than cephalic papillae vs. labial papillae and cephalic papillae of equal size, buccal cavity length [(34–46 vs. 48–54 µm (Winiszewska and Susulovsky, 2003) vs. 39–64 µm Ahmad and Jairajpuri, 2010)], dimensions of buccal cavity less than 1.7 times as long as wide vs. more than 1.8 times as long as wide, amphid aperture position (just above tooth apex vs. at level of anterior end of buccal cavity), position of dorsal tooth from buccal cavity base 75–81% vs. 72–76% (Cobb, 1916), oviduct-uterus constriction moderate vs. no constriction (Ahmad and Jairajpuri, 2010; Clark, 1960) or vs. strong constriction (Winiszewska and Susulovsky, 2003), *pars proximalis vaginae* with almost straight walls vs. straight walls, *pars refringens vaginae* sclerotization surface smooth vs. granular (Winiszewska and Susulovsky, 2003), tail sickle-shaped vs. conoid to ventrally arcuate, and male absent vs. present.

### Molecular characterization

During the survey, two sequences for each gene were generated. However, since they were identical only one sequence of 18 S rDNA (accession number MW218002, 848 bp long) and one sequence of 28 S rDNA (accession number MW227656, 730 bp long) were selected for phylogenetic analyses. A BLASTn search for matches to the partial 18 S rDNA sequence of *P. jonkershoekensis* n. sp. revealed 99% similarity to *P. muscorum* (accession number AJ966500) and 98% similarity to *Coomanus parvus* Jairajpuri & Khan, 1977 (accession number AY284767). Based on 28 S rDNA sequence, the new species only showed 92% similarity to an unidentified isolate of *Prionchulus* (accession number KY750805) and 91% similarity to *P. punctatus* (accession number MG994945).

### Phylogenetic relationships

The 18 S alignment was prepared using 47 sequences (739 nt long) in which 223 nt were variable, while the 28 S alignment contained 23 sequences (760 nt long), of which 494 nt were variable.

**Figure 4:**
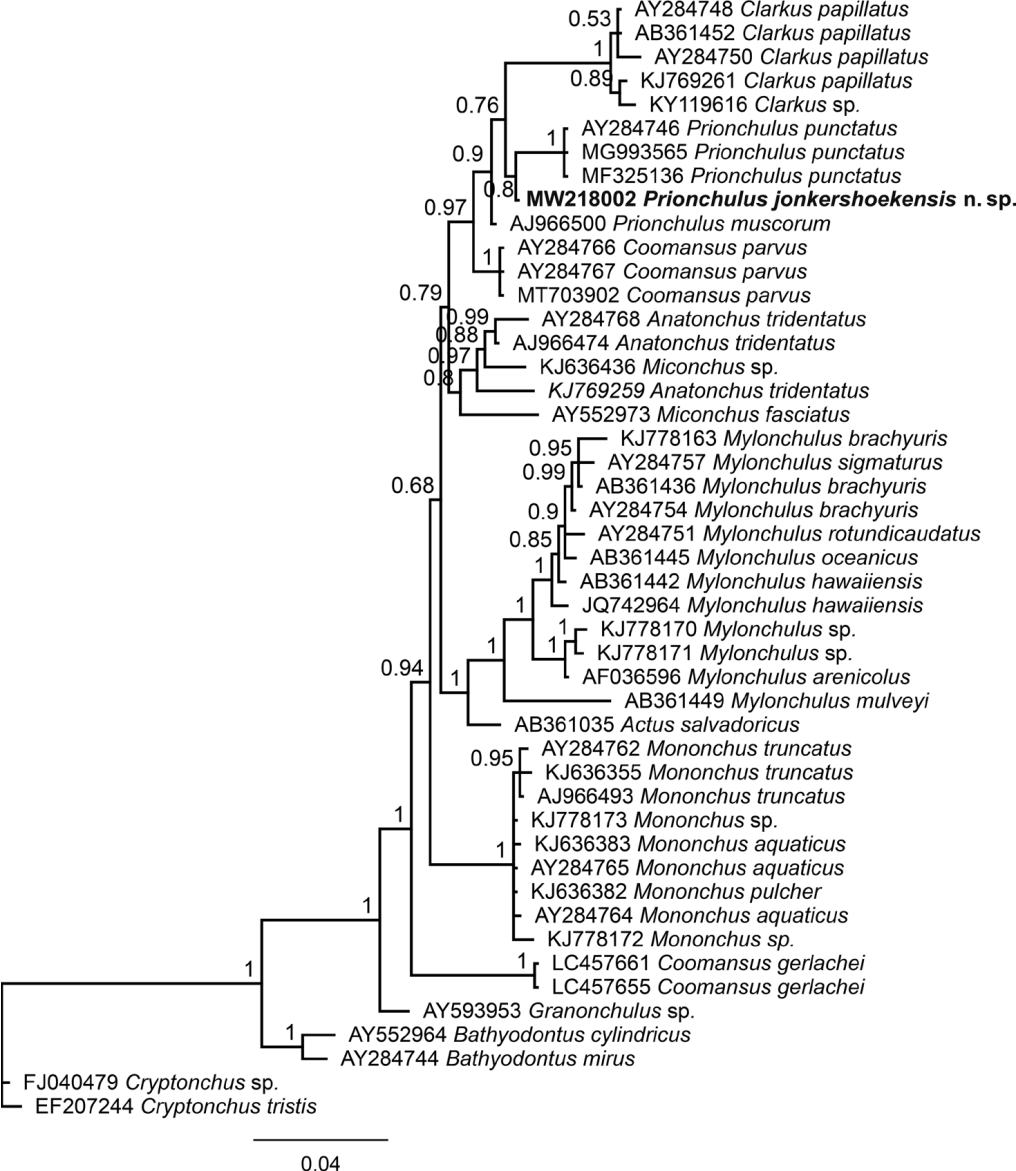
A 50% majority rule Bayesian phylogenetic tree of Mononchidae, including *Prionchulus jonkershoekensis* n. sp. from South Africa, based on the partial 18 S rDNA sequences under the GTR + G model. The sequence of the new species is in boldface font.

**Figure 5:**
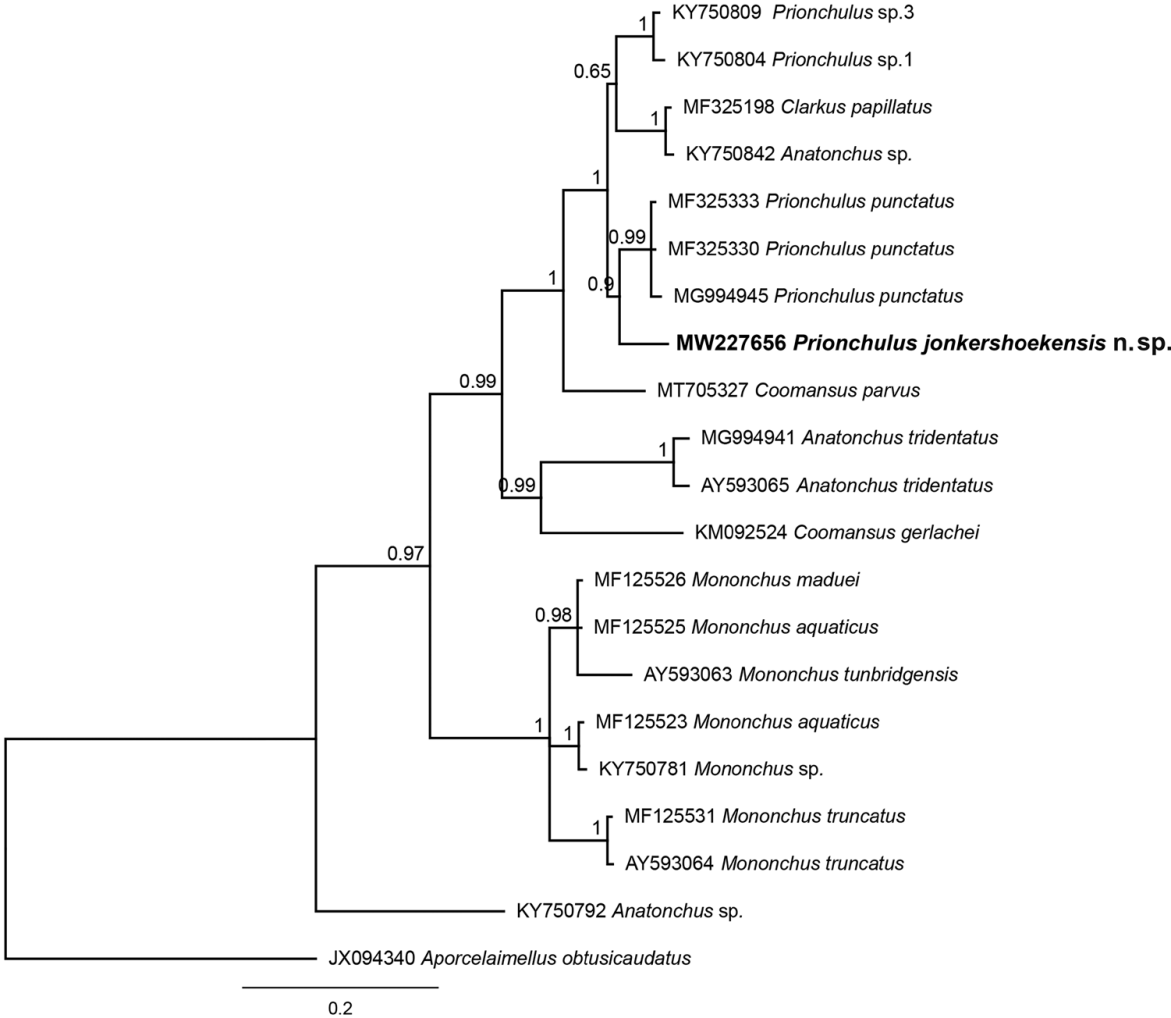
A 50% majority rule Bayesian phylogenetic tree of Mononchidae, including *Prionchulus jonkershoekensis* n. sp. from South Africa, based on the partial 28 S rDNA sequences under the GTR + G model. The sequence of the new species is in boldface font.

Bayesian inference analyses using partial 18 S rDNA sequences clustered *P. jonkershoekensis* n. sp. closely with populations of *P. punctatus* and *P. muscorum* (Fig. 4). The 28 S rDNA Bayesian tree showed the new species forms a supported clade (BPP: 0.9) with three populations of *P. punctatus* (Fig. 5). Interestingly, both phylogenetic trees showed that *Prionchulus* and *Clarkus* Jairajpuri, 1970 are closely related genera.

## Discussion

During this study, a population of *Prionchulus* representing a new species was found in South Africa and described herein as *P. jonkershoekensis* n. sp. using morphological, morphometrical, and molecular approaches. This also represents an additional geographical record for the genus *Prionchulus*. Two notable traits of the new species are its smaller buccal cavity dimensions and lower position of dorsal tooth. The phylogenetic position of *P. jonkershoekensis* n. sp. was resolved using sequences of partial 18 S and 28 S rDNA genes, however the monophyly of the genus *Prionchulus* was not established based on either of these genes. Although the new species shares some similarities with *P. muscorum*, phylogenetic analyses showed that this species and *P. jonkershoekensis* n. sp. are clearly distinct (Table 1).

**Table 1.** Morphometric data of *Prionchulus jonkershoekensis* n. sp.

	Female	
Characters	Holotype	Paratypes
n	–	24
L	1.84	1.97 ± 0.1 (1.78–2.14)
a	23	26.9 ± 1.8 (23.0–29.7)
b	3.1	3.3 ± 0.2 (3.0–3.5)
c	11.6	13.0 ± 0.7 (11.6–14.3)
c’	3.3	3.2 ± 0.2 (2.9–3.5)
V	67	63.3 ± 2.6 (58.6–67.4)
Lip region height	9	9.8 ± 1.3 (8.0–12.0)
Lip region width	37	37.0 ± 2.4 (32.0–40.0)
Amphid from anterior end	14.4	14.6 ± 1.7 (11.2–18.0)
Buccal cavity length	42	41.6 ± 1.4 (38.0–44.0)
Buccal cavity width	26	27.2 ± 1.9 (24.0–31.2)
Position of tooth apex (%)	29	28.5 ± 2.4 (25.0–33.0)
Nerve ring to anterior end	195	184 ± 5.0 (175–195)
Secretory-excretory pore to anterior end	220	206 ± 14.0 (185–220)*
Neck length	600	597 ± 10.9 (580–610)
Cardia length	11.2	12.3 ± 1.3 (10.0–15.0)
Cardia width	36	35.0 ± 2.6 (30.0–40.0)
Cuticle – lip region	2.0	1.8 ± 0.3 (0.8–2.4)
Cuticle – at vulva	2.4	2.8 ± 0.9 (2.0–4.0)
Cuticle – at tail	4	3.0 ± 1.0 (2.0–4.0)
Lateral chord width	22	25 ± 2.4 (20.0–28.0)
Body diameter – neck base	67	67.2 ± 2.7 (64.0–75.0)
Body diameter – at midbody	80	73.5 ± 4.3 (66.0–82.0)
Body diameter – at anus	48	46.6 ± 2.4 (44.0–52.0)
Vagina length	26	31.2 ± 2.9 (26.0–35.0)
Rectum length	36	40.1 ± 3.1 (36.0–48.0)
Tail length	158	152 ± 4.7 (144–158)
Hyaline length	6	6.8 ± 1.7 (4.0–8.0)
Egg length	95	84.0 ± 7.5 (76.0–95.0)**
Egg width	60	45.0 ± 9.0 (37.0–60.0)**

Notes: ^*^n = 14. ^**^n = 8. All measurements are in μm (except L, in mm), and in the form: mean ± standard deviation (range).
